# Prophylactic Antibiotics to Prevent Cellulitis of the Leg: Economic Analysis of the PATCH I & II Trials

**DOI:** 10.1371/journal.pone.0082694

**Published:** 2014-02-14

**Authors:** James M. Mason, Kim S. Thomas, Angela M. Crook, Katharine A. Foster, Joanne R. Chalmers, Andrew J. Nunn, Hywel C. Williams

**Affiliations:** 1 Durham Clinical Trials Unit, Durham University, Queen’s Campus, Stockton-on-Tees, United Kingdom; 2 Centre of Evidence-Based Dermatology, University of Nottingham, Nottingham, United Kingdom; 3 Medical Research Council Clinical Trials Unit, London, United Kingdom; Johns Hopkins Bloomberg School of Public Health, United States of America

## Abstract

**Background:**

Cellulitis (erysipelas) is a recurring and debilitating bacterial infection of the skin and underlying tissue. We assessed the cost-effectiveness of prophylactic antibiotic treatment to prevent the recurrence of cellulitis using low dose penicillin V in patients following a first episode (6 months prophylaxis) and more recurrent cellulitis (12 months prophylaxis, or 6 months in those declining 12 months).

**Methods:**

Within-trial cost-effectiveness analysis was conducted using the findings of two randomised placebo-controlled multicentre trials (PATCH I and PATCH II), in which patients recruited in the UK and Ireland were followed-up for up to 3 years. Incremental cost, reduction in recurrence, cost per recurrence prevented and cost/QALY were estimated. National unit and reference costs for England in 2010 were applied to resource use, exploring NHS and societal perspectives. A total of 397 patients from the two trials contributed to the analysis.

**Results:**

There was a 29% reduction in the number of recurrences occurring within the trial (IRR: 0.71 95%CI: 0.53 to 0.90, p = 0.02), corresponding to an absolute reduction of recurrence of 0.31 recurrences/patient (95%CI: 0.05 to 0.59, p = 0.02). Incremental costs of prophylaxis suggested a small cost saving but were not statistically significant, comparing the two groups. If a decision-maker is willing to pay up to £25,000/QALY then there is a 66% probability of antibiotic prophylaxis being cost-effective from an NHS perspective, rising to 76% probability from a secondary, societal perspective.

**Conclusion:**

Following first episode or recurrent cellulitis of the leg, prophylactic low dose penicillin is a very low cost intervention which, on balance, is effective and cost-effective at preventing subsequent attacks. Antibiotic prophylaxis reduces cellulitis recurrence by nearly a third but is not associated with a significant increase in costs.

## Introduction

Cellulitis (erysipelas) of the leg is a common infection of the skin and sub-cutaneous tissue that recurs in up to 50% of cases [1], and is most commonly caused by streptococcal infection [2], [3]. Recurrent episodes lead to progressive damage of the lymphatic system, resulting in lymphedema and increased risk of recurrence and associated costs of care [4], [5]. While antibiotic treatment of an acute episode of cellulitis is uncontroversial [6], physicians’ views on whether low dose antibiotics should be used to prevent cellulitis recurring are variable.

This paper describes a within-trial health economic analysis of two randomised controlled trials addressing the prevention of cellulitis, using low dose prophylactic penicillin (PATCH I and PATCH II) [7], [8]. Prior to these trials, the evidence base was limited to three small inconclusive randomised controlled trials (120 patients in total) published between 1991 and 1994 [9]–[11], with no trial-based economic analysis. Consequently, current guidance on preventing cellulitis is largely consensus based [12], [13]. In England in 2011, limb cellulitis accounted for 52,654 hospital admissions and 373,343 bed days at an approximate cost of £120 million [14], [15]. Hospitalised patients represent the most severe cases, although many patients are treated in the community. A study in the Netherlands in 2001, found that only 7% of all patients were hospitalized although 83% of the total treatment costs occurred in hospital [16].

In brief, the PATCH I and II trials were double-blind, parallel group, randomised controlled trials comparing low dose penicillin V (250 mg bd) with matched placebo [used prophylactically] to prevent recurrence of cellulitis of the leg (PATCH I: ISRCTN34716921 and PATCH II: ISRCTN03813200) [7], [8]. Recruitment occurred in 28 hospitals in the UK and Ireland between June 2006 and January 2010. Potential participants were identified for the trials if they had suffered a recent episode of cellulitis of the leg; they were consented and randomised to prophylaxis with penicillin or placebo after treatment of the acute episode had been completed. Both trials followed patients for up to three years. PATCH I recruited 274 patients with recurrent cellulitis who were given 12 months prophylaxis, while PATCH II recruited 123 patients who were given six months prophylaxis following a first episode of cellulitis (n = 97) or those with recurrent cellulitis who refused 12 months of prophylaxis (n = 26). PATCH I reported a reduced risk of first recurrence during the prophylaxis phase to 12 months (hazard ratio, HR 0.55, 95% CI 0.35, 0.86; p = 0.01), with 22% of treated patients having a repeat episode compared with 37% in the placebo group. PATCH II reported a reduced risk of first recurrence over the full three year period of follow-up (hazard ratio, HR: 0.53, 95%CI: 0.26 to 1.07, P = 0.08], with 20% of treated patients having a repeat episode compared with 33% in the placebo group.

To inform policy and clinical practice about the cost-effectiveness of prophylaxis to prevent recurrent cellulitis, an economic analysis was planned prospectively within the PATCH trial designs.

## Methods

Within-trial patient-level cost-effectiveness analyses were undertaken using data from the PATCH I and II trials. Patient data were analysed using the same intention to treat principles applied to the clinical outcomes. The primary analysis was from the NHS perspective with a secondary societal analysis. Because of the similarity of the methods, outcomes and results in the two trials a patient level analysis was conducted combining the participants of both PATCH I and II.

### Outcomes

Within the clinical trial reports the primary outcome of interest was time to first recurrence with the relative rate between groups expressed as a hazard ratio. However the most important outcome from an economic perspective is the number (or count) of recurrences, hence numbers of recurrences are set against the costs of care, which include management of recurrent cellulitis and antibiotic prophylaxis (in the active treatment arm). First and subsequent recurrences were recorded in both trials using the same clinical reporting mechanism and all were clinically verified as described within the trial reports [7], [8].

Quality of life measures included the EuroQoL EQ-5D-3L [17] and Dermatitis Life Quality Index, DQLI [18]. The EuroQoL EQ-5D-3L is a generic quality of life measure with 5 domains each scored at 3 levels: findings are mapped onto societal health state preference values referenced to scores of 0 (dead) and 1 (perfect health). The Dermatology Life Quality Index (DLQI) is a disease specific quality of life measure including 10 questions each with 4 levels (0–3) scored from 0 (no effect) to 30 (extremely large effect on quality of life). Although we attempted to collect quality of life data during the trial at the time of recurrences, this was often unsuccessful due to a time lag in being notified of the event. However these measures were assessed for patients during and after resolution of their index infection at baseline (before commencement of trial prophylaxis). From these data, the reduction in DLQI and quality-adjusted life-years due to a recurrence were estimated, comparing measures to demonstrate consistency. The QALY loss due to recurrence at baseline was used to model QALY gains due to recurrence prevention during the trial phase. Effective treatment normally makes cellulitis a short-lived event lasting 7–10 days [4]. As a result, the impact on participants’ quality of life was estimated using index episode data, providing a QALY ‘tariff’ for a recurrence.

### Resource Use and Cost

Patient diaries were used to capture numbers of days spent in hospital, outpatient visits, community nurse contacts, GP consultations, prescriptions and time off work or away from routine activities related to the treatment of cellulitis and its sequelae (e.g. ulcers or lymphedema). These were then reported to the trial team during scheduled telephone follow-up contacts (at 3 or 6 monthly intervals depending on the stage of the trial). Recurrent episodes were confirmed by clinical consultation with either the study investigator or the participant’s general practitioner at the time of the recurrence. Episodes that were unable to be confirmed by a medical professional were excluded from the analysis. National unit costs for 2010 were applied to resources providing a cost of care for each patient during follow-up. Outpatient visits, community nurse contacts and GP consultations were costed at £152, £27 and £36 per item respectively [15]. Hospital consultations were costed at £319/day - the reference cost [19] for intermediate skin disorders category two obtained by mapping the ICD10 code for cellulitis (L03) to the HRG code JD03 [20]. Prescriptions were costed using prices provided in the British National Formulary [6]. Time away from work or normal activities was costed at £110/day using the Annual Survey of Hours and Earnings (ASHE) [21]. The societal perspective included all reported cost items; costs of time away from work or normal activities were excluded for the NHS perspective.

### Analysis

Although prophylaxis periods were 12 months and 6 months in the PATCH I and PATCH II trials respectively, a common treatment period of 12 months (with post treatment of up to 24 months) was used to structure the data for comparability and analytic convenience. Since cost data and recurrence data are highly skewed, treatment group differences and confidence intervals were estimated stochastically using bootstrap methods with 1,000 replications per estimate (i.e. resampling the patient-level data with replacement) [22]. For counts of recurrence, incident rate ratios were estimated conservatively using a generalised linear model using the negative binomial with log link function. Cost-effectiveness planes were generated and used to generate cost-effectiveness acceptability curves (CEACs) using standard methods [23]. The CEAC, derived from the joint distribution of costs and effects, illustrates the (Bayesian) probability that the data are consistent with an underlying (true) level of cost-effectiveness, precluding a simple frequentist interpretation of probability values. The primary analysis included combined patient-level from both the PATCH I and II trials. The effect of discounting costs and benefits was explored using discount rates of 0%, 3.5% and 5% with the primary analysis estimating future costs and benefits discounted at 3.5%, in line with NICE Guidance [24]. In addition to discounting, the influence of outlier values, analyses of individual trial findings and a model of continuous prophylaxis were explored in sensitivity analyses. Analyses were performed in SPSS 21 © 2012 IBM Corporation and Excel v14 © 2010 Microsoft Corporation.

## Results

### Patient Characteristics

The characteristics of patients recruited into the PATCH I and II trials have been previously reported. In brief, there were no differences between groups in either trial at baseline. PATCH I patients were: median age 58 (IQR 48–67); 66% female; BMI 33 (IQR: 28–40); 100% had a history of recurrent cellulitis, 68% had pre-existing oedema, ulcer or both. PATCH II patients were: median age 59 (IQR 48–71); 66% female; BMI 31 (IQR: 27–36); 21% had a history of recurrent cellulitis, 51% had pre-existing oedema, ulcer or both.

### Recurrence

**Figure 1:**
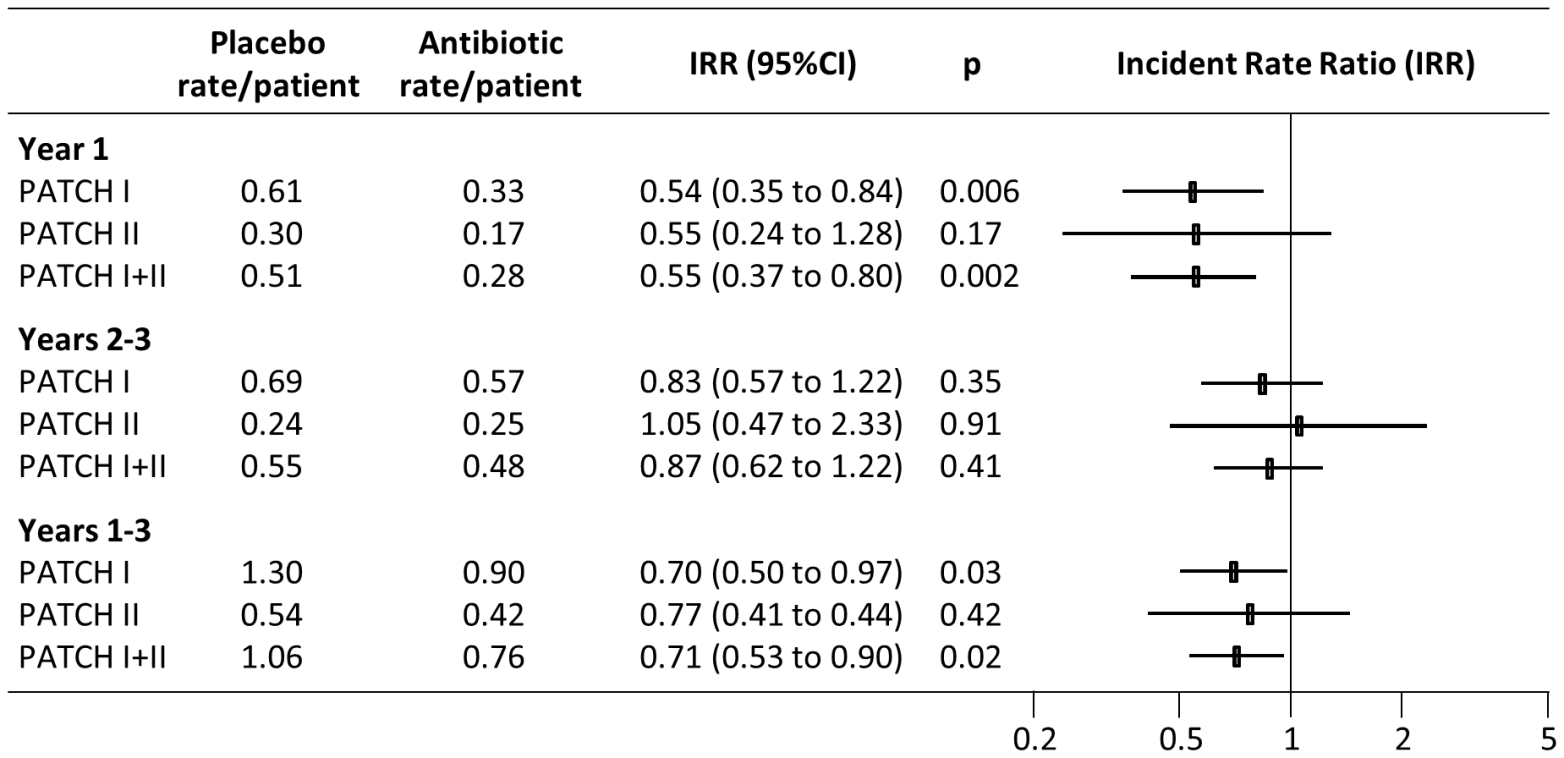
Analysis of recurrence rates in the PATCH trials, exploring on-prophylaxis, post-prophylaxis and overall rates.

There was a consistent pattern of relative benefit in the two trials over 3 years of follow-up, although findings from PATCH II did not reach statistical significance, due to limited power. The relative rate of recurrence expressed as the incident rate ratio (IRR) was consistent between trials showing similar benefit in the first year but no persisting protective effect in years 2 and 3 (see Figure 1). Overall there was a 29% reduction in the number of recurrences occurring within the trial (IRR: 0.71, 95%CI: 0.53 to 0.90, p = 0.02), corresponding to an absolute reduction of recurrence of 0.31 recurrences/patient (95%CI: 0.05 to 0.59, p = 0.02).

### Quality of Life

EQ-5D and DLQI scores were obtained during a screening visit and again at 10 days post randomisation for a random subsample of patients: complete data from both periods were available for 200 patients. At the screening visit, participants were categorised as either having an ongoing episode of cellulitis (n = 71), or having had a recent episode of cellulitis that had subsequently resolved (n = 129). Consequently EQ-5D and DLQI scores could be compared in patients during and after infection with a comparison group with no contemporaneous infection. Table 1 shows similar scores when comparing screening and 10 day findings in the no infection group but significant improvement in the patients undergoing treatment for infection. Applying an average of 7 days infection and a 26.3% reduction in quality of life, a recurrence is approximately worth 0.263×7/365 of 0.005 QALYS. (This is taken as approximately equivalent to a infection time-varying in severity over ten days where quality of life improves once antibiotic therapy takes effect). Consequently a reduction of 0.305 recurrences/patient is worth approximately 0.0015 QALYs. Qualitatively DLQI scores provided a similar finding with no clinically significant differences in the two periods for the no infection group but improvement by nearly 10 points in the infection group.

**Table 1:** Effect of cellulitis on quality of life.

	t_0_ *		t_1_ *					
	mean	(sd)	mean	(sd)	N	t_1_–t_0_	(95%CI)	p
EQ-5D								
No Infection+	0.733	(0.264)	0.746	(0.296)	125	0.013	(−0.025 to 0.051)	0.50
Infection#	0.436	(0.342)	0.699	(0.291)	70	0.263	(0.180 to 0.352)	<0.001
DLQI								
No Infection+	5.15	(5.51)	3.72	(4.74)	129	−1.43	(−2.31 to −0.54)	0.001
Infection	14.11	(8.09)	4.56	(4.81)	71	−9.55	(−11.50 to −7.83)	<0.001

*Baseline trial visits: t_0_ = screening visit, t_1_ = trial visit at day 10.

+ Patient with no infection at t_0._

# Patient with infection at t_0._

### Costs Analysis

Resource items contributing to costs are reported in Table 2. No resource items differed significantly comparing antibiotic and placebo groups for the combined trials or for the individual trials. Unit costs were applied to resources use and together with drug costs provided overall NHS treatment costs. Societal costs included a valuation of time away from normal activities. Consistent with the resource data there were no significant NHS or societal cost differences between groups.

**Table 2:** Resource use, cost and outcome in the combined PATCH trials.

	Antibiotic,	N = 196	Placebo,	N = 201			
	Mean	(SD)	Mean	(SD)	Δ*	(95% CI )	p
GP visits	3.41	(17.90)	2.84	(11.64)	0.57	(−2.20 to 3.68)	0.76
Community nurse visits	6.61	(44.32)	2.13	(14.10)	4.48	(−1.08 to 11.64)	0.22
Inpatient stays	0.16	(0.56)	0.15	(0.55)	0.00	(−0.11 to 0.11)	0.94
Inpatient days	0.98	(3.66)	2.06	(15.25)	−1.08	(−3.59 to 0.53)	0.46
Outpatient visits	0.41	(1.63)	0.61	(3.04)	−0.19	(−0.73 to 0.23)	0.46
Days off work	3.17	(14.98)	5.10	(19.61)	−1.94	(−5.38 to 1.33)	0.27
Drugs cost+							
Study, r_c_ = 0%	29.82	−	0.00	−	29.82	(28.63 to 30.95)	<0.001
Non-study, r_c_ = 0%	16.22	(65.32)	18.23	(55.86)	−2.01	(−14.49 to 9.80)	0.76
NHS treatment cost+							
r_c_ = 0%	722	(2090)	927	(4931)	−205	(−1049 to 402)	0.63
r_c_ = 3.5%	704	(2034)	900	(4768)	−197	(−1009 to 387)	0.63
r_c_ = 5%	696	(2011)	889	(4702)	−193	(−992 to 384)	0.63
Societal Cost+							
r_c_ = 0%	1069	(2750)	1486	(6437)	−417	(−1479 to 383)	0.48
r_c_ = 3.5%	1047	(2698)	1452	(6240)	−406	(−1440 to 375)	0.48
r_c_ = 5%	1038	(2678)	1439	(6159)	−401	(−1426 to 371)	0.48
Recurrence rate							
r_c_ = 0%	0.755	(1.278)	1.060	(1.434)	−.305	(−0.588 to −0.050)	0.02
r_c_ = 3.5%	0.732	(1.233)	1.034	(1.394)	−.303	(−0.575 to −0.054)	0.02
r_c_ = 5%	0.722	(1.215)	1.024	(1.378)	−.302	(−0.572 to −0.056)	0.02

*Δ: Antibiotic – Placebo.

+ 2010 costs; r_c_ refers to the discount rates applied to costs distributed over time.

### Incremental Cost-Effectiveness

**Figure 2:**
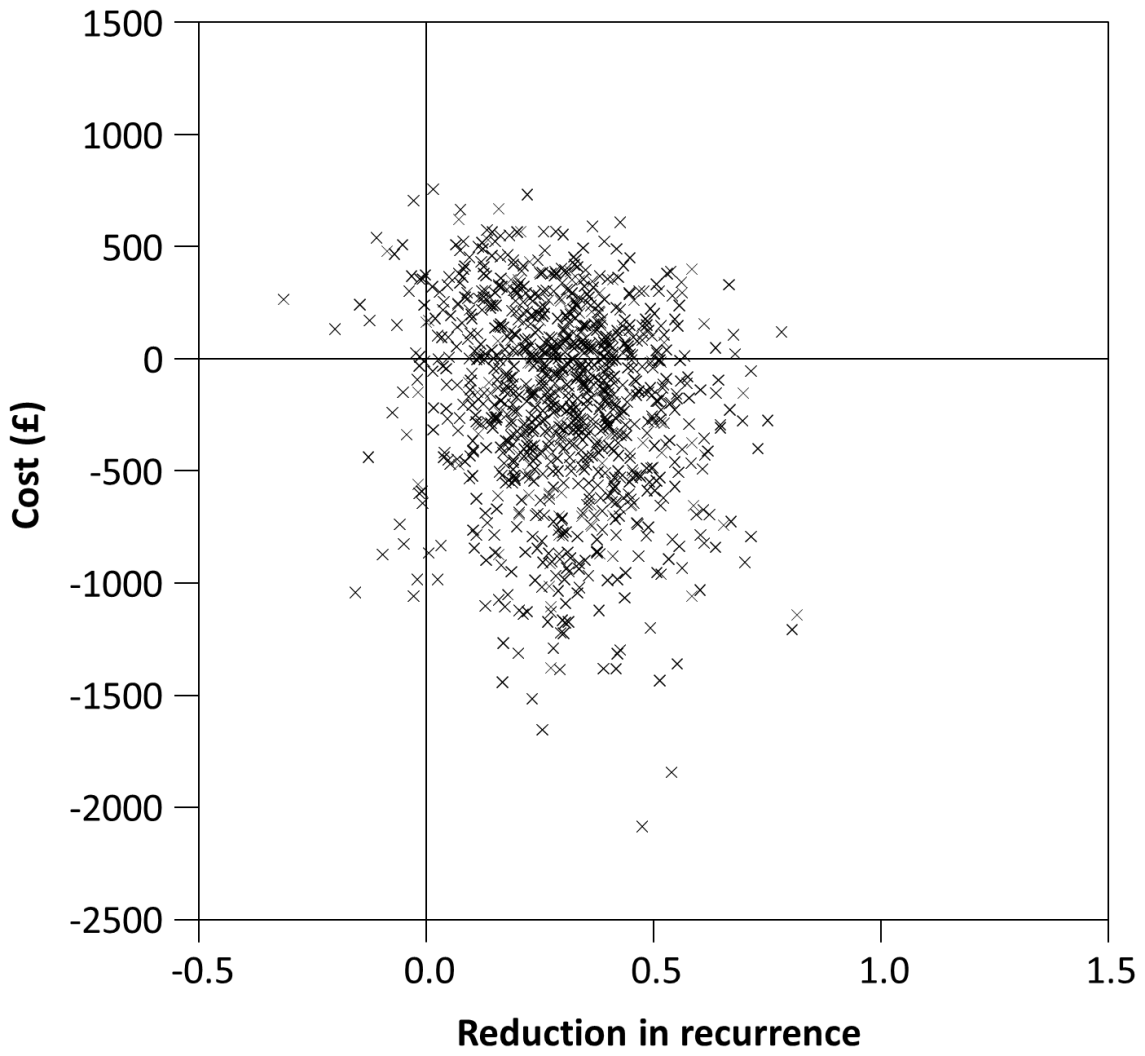
Cost-effectiveness plane (NHS costs and benefits discounted at 3.5%).

Patient-level data were bootstrapped and visualised on the cost-effectiveness plane (Figure 2, NHS costs). The distribution of replicates shows a mean cost saving of £197 and reduction in recurrence of 0.303 per patient, with future costs and benefits discounted at 3.5%. Thus on average antibiotic prophylaxis was cost saving while reducing recurrences.

**Figure 3:**
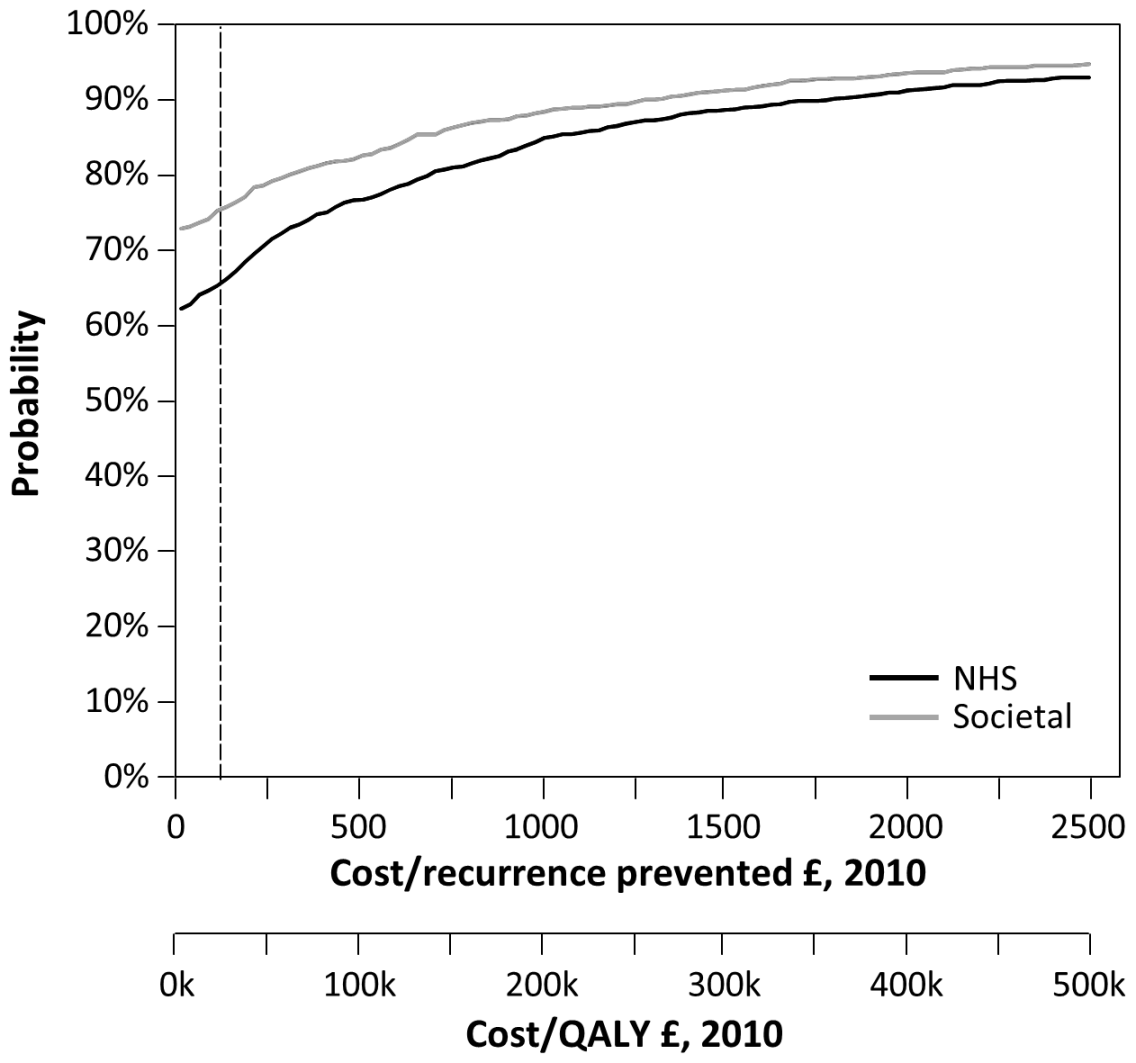
Cost-effectiveness acceptability curves (CEACs), NHS and Societal costs and benefits discounted at 3.5%.

The cost-effectiveness acceptability curve (CEAC) was generated (the proportion of replicates to the south and west as a line sweeps anticlockwise from the x-axis to the y-axis on the cost-effectiveness plane) and is shown in Figure 3. The CEAC indicates the probability that prophylaxis is cost-effective for a range of cost-effectiveness ratios values. The two x-axes show cost per recurrence and cost/QALY. If a decision-maker is willing to pay up to £25,000/QALY (shown as a dashed line, the middle of the range £20–30K/QALY used in England by NICE as assess value for money [24]), then there is a 66% probability of this being true from an NHS perspective and 76% probability from a secondary, societal perspective. Investing for lower returns (or a higher cost/QALY), it becomes increasingly probable that antibiotic prophylaxis is cost-effective. If a break-even (no-investment) criterion is required then prophylaxis is 62% likely to be cost-effective from an NHS perspective and 73% from a secondary, societal perspective.

### Discounting

Discounting had little effect upon any of the incremental estimates of cost, effect or cost-effectiveness estimates reported (see Table 2).

### Sensitivity Analysis

Sensitivity analyses included the influence of outlier values, separate analyses of the PATCH I and II trials and modelling of continuous prophylaxis.

#### Individual trials and outlier values

**Figure 4:**
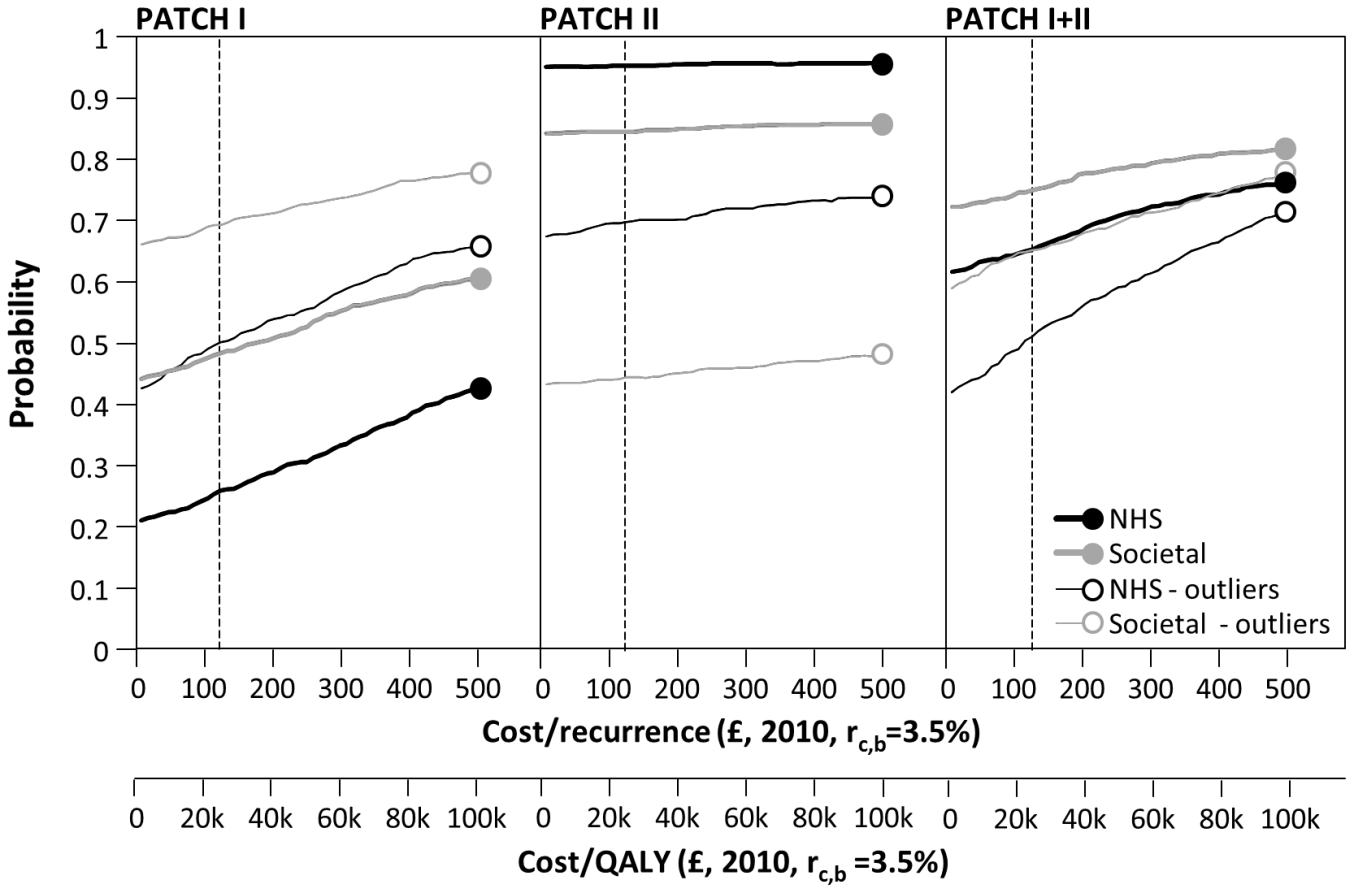
Sensitivity Analyses exploring influence of cost outliers and individual trials, NHS and Societal costs and benefits discounted at 3.5%.

Four patients within the two trials had very high, cellulitis-related costs during the trial and there was evidence of raised health service usage before enrolment in these patients. Two such patients were present in the placebo group of PATCH II and two in the antibiotic group in PATCH I, thus apparently increasing the cost-effectiveness of PATCH II and reducing the cost-effectiveness of PATCH I. Figure 4 shows the individual trial CEACs with and without the outlier patients: the individual trial findings converge to the pooled finding for PATCH 1 and II. Removal of further higher cost patients or high recurrence patients from either trial had no qualitative or systematic impact on findings.

#### Continuous prophylaxis

There was a more dramatic 45% reduction in the number of recurrences while on prophylaxis (incident rate ratio, IRR: 0.55 95%CI: 0.37 to 0.80), p = 0.002 (see Figure 1). It was possible to approximate continuous prophylaxis by modelling the cost and recurrence data to one year and excluding subsequent post-treatment data. The assumption made is that benefit and cost profiles within the first year are stable and persistent. Modelling one-year data produced virtually identical findings to the main analysis, which might be expected since the net costs and benefits after one year are not significantly altered after three years.

## Discussion

### Main Findings

Antibiotic prophylaxis substantially reduced the number of recurrences experienced by patients while on treatment although there was no evidence of a persisting protective effect when therapy ceased. There was no evidence of a ‘rebound’ with a compensating greater frequency of cellulitis after cessation of antibiotic treatment, thus there may be a case for extending antibiotic treatment to prolong benefit, as occurs in current clinical practice in the management of individual patients.

Antibiotic prophylaxis as a policy for treatment following either first episode or recurrent cellulitis is likely to be cost-effective to the NHS, although CEAC analysis shows this finding is on balance of probability (66%) rather than with conventional of statistical certainty (90% or 95%). This finding is largely a consequence of large variations in patient costs, leading to imprecise cost-effectiveness estimates. Consequently, the incremental cost effectiveness of penicillin prophylaxis couples a precise estimate of benefit (98% probability of net benefit) with greater cost uncertainty (62% probability of net cost savings). Notwithstanding these uncertainties, these two trials together provide the best evidence currently available to explore a policy of prophylaxis (tailored by duration of treatment) to first episode and recurrent cellulitis.

While post hoc secondary analysis of the PATCH I trial confirmed that patients with a high BMI, multiple previous episodes and/or lymphedema of the leg had substantially greater risk of recurrence, there was no interaction with the efficacy of treatments. Overall findings for number of recurrence (not tabulated) similarly showed no significant interaction between these variables and treatment. Thus there is currently no evidence to target prophylaxis to patients on the basis of these risk profiles [25].

### Strengths and Weaknesses

The economic analysis of the PATCH I and PATCH II was planned according to a prospectively defined protocol in which these trials were to be analysed separately. However PATCH II under-recruited, and cost data from both trials was imprecise (reducing the ability of the individual trial economic analyses to be informative). Comparison of recurrence and cost findings provided a valid basis for a combined analysis. Consequently the economic analysis addresses the policy relevant question of should antibiotic prophylaxis (of appropriate duration) be offered to patients following first episode or recurrent cellulitis. When conducting a patient level analysis of the two trials combined, the analysis maintains the protection against bias provided within the randomised controlled design. However, this does provide an arbitrary weighting of overall findings, reflecting the numbers of patients in the two trials.

Patients were asked to collect and report information on cellulitis-related resource use. Thus there was a risk of under-reporting, although the randomised design provides some protection against systematic differences between groups.

The value of preventing an episode of cellulitis in terms of QALYs gained was approximated using screening and baseline DLQI scores in those with and without ongoing cellulitis at the time of trial recruitment. The QALY estimate is likely to be conservative, since some patients may have already been recovering at the screening visit and some patients may have had ongoing infection at the baseline visit, typically ten days later. Thus it is possible that the likelihood of cost-effectiveness has been underestimated although this would not alter the findings of the analysis qualitatively. By inspection of Figure 3, a doubling of the QALY gain per recurrence would increase the likelihood of cost-effectiveness at a threshold of £25,000 from 66% to 72%.

### Generalizability

PATCH I and II were pragmatically designed to reflect normal clinical care, with minimal contact between patients and recruiting clinicians. Trial design features (e.g. appropriately generated randomisation, use of double-blinding and low dropout rates) are likely to have minimised bias. Baseline characteristics of trial participants were representative of the spread of patients routinely seen in the UK and Ireland. These results should generalise well to patients in countries other than the UK with similar healthcare systems and where overall levels of hospitalisation are low.

### Implications for Practice

Economic analysis of the PATCH trials suggests that a policy of antibiotic prophylaxis is likely, on balance, to be cost-effective for patients both with first episode and recurrent cellulitis. The clinical implication is that antibiotic prophylaxis reduces cellulitis recurrence by nearly a third and is not associated with a significant increase in cost.
